# Association between serum testosterone and measures of cardiovascular health among transgender individuals using gender-affirming testosterone therapy: a cross-sectional study

**DOI:** 10.1186/s13293-025-00726-3

**Published:** 2025-06-17

**Authors:** Badal S. B. Pattar, Tyrone G. Harrison, Nathalie Saad, Sandra M. Dumanski, A.J. Lowik, Paul E. Ronksley, Dina N. Greene, Cameron T. Whitley, Chantal L. Rytz, Keila Turino Miranda, Lindsay Peace, Amelia M. Newbert, Darlene Y. Sola, Sofia B. Ahmed

**Affiliations:** 1 Cumming School of Medicine, University of Calgary, Calgary, AB Canada; 2 Libin Cardiovascular Institute, University of Calgary, Calgary, AB Canada; 3 O’Brien Institute for Public Health, University of Calgary, Calgary, AB Canada; 4 Department of Medicine, University of Calgary, Calgary, AB Canada; 5 Department of Community Health Sciences, University of Calgary, Calgary, AB Canada; 6 Department of Sociology, University of Lethbridge, Lethbridge, AB Canada; 7 Department of Laboratory Medicine, University of Washington, Seattle, Washington, USA; 8 Department of Sociology, Western Washington University, Bellingham, Washington, USA; 9 Cardiovascular Health and Autonomic Regulation Laboratory, Department of Kinesiology and Physical Education, McGill University, Montreal, QC Canada; 10 Skipping Stone Foundation, Calgary, AB Canada; 11 Department of Medicine, Faculty of Dentistry and Medicine, University of Alberta, Edmonton, AB Canada; 12 Women and Children’s Health Research Institute, University of Alberta, Edmonton, AB Canada

**Keywords:** Transgender, Gender-affirming hormone therapy, Testosterone, Serum testosterone, Cardiovascular health, Blood pressure, Arterial stiffness

## Abstract

**Background:**

Gender-affirming testosterone therapy (GATT) use may be associated with increased systolic blood pressure (SBP). The association between serum testosterone and cardiovascular health in individuals using GATT is unknown. The objective of this study was to estimate the association between serum testosterone and validated measures of cardiovascular health, including SBP and arterial stiffness, in persons assigned female sex at birth using GATT.

**Methods:**

Healthy participants assigned female sex at birth on a stable GATT regimen for ≥ 4 months were recruited to this community-partnered exploratory cross-sectional study. Exposures of interest were total and free serum testosterone concentration. As our primary outcome, SBP was measured by an automated sphygmomanometer, and carotid-radial pulse wave velocity (PWVcr) and aortic augmentation index (AIx) were used to measure arterial stiffness via applanation tonometry.

**Results:**

Participants (n = 18, median age 28 years, range: 18, 50) who predominantly self-identified as white (94%) and had been using GATT for a median of 48 months (range: 5, 84) were studied. Resting SBP, PWVcr, and AIx were 113 mmHg (range: 102, 129), 7 m/s (range: 4, 9), and 9% (range: − 10, 23), respectively. Total and free serum testosterone were not significantly associated with SBP or PWVcr. Free, but not total, serum testosterone was positively associated with AIx (p = 0.03). Sensitivity analyses did not modify any results.

**Conclusions:**

In healthy transgender individuals, serum testosterone concentrations may not be associated with measures of cardiovascular health. However, these results need to be interpreted with caution given the limited sample size.

**Supplementary Information:**

The online version contains supplementary material available at 10.1186/s13293-025-00726-3.

## Highlights

- We assessed the association between serum testosterone concentration and markers of cardiovascular health in transgender individuals using GATT.
- We did not identify an association between serum total testosterone concentrations and cardiovascular health markers in healthy transgender individuals treated with GATT.
- In healthy transgender individuals treated with GATT, serum testosterone concentrations may not be associated with measures of cardiovascular health.
- Although longitudinal studies are required, these results may inform shared decision-making between individuals using GATT and their healthcare providers.

**Supplementary Information:**

The online version contains supplementary material available at 10.1186/s13293-025-00726-3.

## Abstract (plain language summary)

Testosterone therapy is an important part of gender-affirming care for many transgender individuals. However, concerns exist about how this therapy might affect heart and blood vessel health. This study aimed to explore whether testosterone levels in the blood are linked to these measures of cardiovascular health. We studied 18 adults who were assigned female at birth and had been on stable testosterone therapy for at least four months. We measured their blood pressure and used specialized tools to assess arterial stiffness, a marker of blood vessel health. Our results demonstrated that testosterone levels were not significantly linked to blood pressure. Overall, in healthy transgender individuals treated with testosterone, serum testosterone measurements may not be linked to blood pressure or blood vessel health. However, further research is required to confirm this. This research highlights the importance of understanding how testosterone therapy affects the heart and blood vessels to ensure the best possible care for those undergoing gender-affirming treatments.

**Supplementary Information:**

The online version contains supplementary material available at 10.1186/s13293-025-00726-3.

## Introduction

The transgender population experiences disproportionately increased cardiovascular risk compared to their cisgender peers [1, 2]. While factors such as gender minority stress, decreased health care access, and discrimination play important roles, systematic reviews have reported limited and low-quality evidence examining the role of gender-affirming testosterone therapy (GATT) on cardiovascular health parameters [3–5]. GATT is used to suppress feminine and/or develop masculine secondary sex characteristics depending on each individual’s goals [6, 7], and can result in an increase in serum testosterone and decrease in fluctuation of estrogen concentrations [8] that may in part also be dependent on the route of administration [9]. While the measurement of serum sex hormones in addition to monitoring health parameters, such as blood pressure, is routine and part of the gender-affirmation process in individuals treated with GATT [6, 7], the association between serum testosterone concentrations and measures of cardiovascular health is unknown.

Previous studies have suggested a role for serum testosterone in cardiovascular risk. To avoid assumptions on our part when discussing others' work, the terminology related to participants' sex/gender is that reported in the cited reference. In men, low serum testosterone concentrations have been associated with increased risk of cardiovascular diseases [10, 11], hypertension [12], and cardiovascular mortality [13–15]. Data are conflicting among women, where significant negative [16–18] and positive [19–23] associations, as well as no associations [24–26] have been demonstrated between serum testosterone concentrations and cardiovascular risk. Notably, women with serum testosterone concentration at or above the 95th percentile demonstrated a significantly higher risk for ischemic heart disease and death compared to those with concentrations in the 10th to 89th percentiles [20]. However, there are important concerns around generalizing data from one population to another [27]; as such, there is an urgent need to improve the inclusion of transgender individuals in cardiovascular health research to develop appropriate evidence-based interventions [28]. Moreover, although current guidelines for the care of transgender individuals undergoing GATT recommend targeting serum testosterone concentrations within the reference ranges for cisgender men [6, 7], the lack of consensus on optimal transgender-specific testosterone concentrations and limited understanding of associated cardiovascular health consequences prompted our study. Specifically, we explored the relationship between serum testosterone and measures of cardiovascular health in persons assigned female sex at birth using GATT.

## Methods

### Persons with living experience-oriented approach

This study focused on a knowledge gap directly identified by transgender individuals and community organizations. Our research team consists of transgender and cisgender people, including community partners, who worked collaboratively on the conceptualization, manuscript framework, and writing of this work. The methodology and reporting for this cross-sectional study followed recommendations from the *Strengthening the Reporting of Observational Studies in Epidemiology* (STROBE) guidelines for reporting observational studies [29] (**Appendix A**).

### Participants

This cross-sectional study was conducted in Calgary, Alberta, Canada, with participant recruitment between July 2022 and September 2024. Participants underwent a medical history, physical examination and laboratory screening. Participants were eligible if they were ≥ 18 years old, assigned female sex at birth, and had been using GATT for ≥ 4 months, which has previously been shown to be the timepoint when blood pressure stabilizes with GATT use [30]. Exclusion criteria were a history of cardio- or cerebrovascular disease (symptoms consistent with myocardial ischemia, or previously documented myocardial ischemia, cardiac arrythmias, congestive heart failure, valve abnormalities, transient ischemic attacks, or stroke), diabetes mellitus (hemoglobin A1C > 6.5%, fasting glucose > 7 mmol/L or use of hypoglycemic agents), chronic kidney disease (estimated glomerular filtration rate (eGFR) < 60 ml/min/1.73m^2^ or urine albumin to creatinine ratio (uACR) > 3 mg/mmol), hypertension (blood pressure > 140/90 mmHg or use of antihypertensive medications), and hyperlipidemia (low-density lipoprotein cholesterol (LDL-c) > 4.5 mmol/L or use of lipid-lowering agents). Participants were recruited using a snowball and convenience sampling strategy in partnership with a community organization [31] and gender-affirming care clinics. Given the exploratory nature of this study, a sample size calculation was not performed.

### Protocol

Participants underwent a structured interview to collect self-reported demographic information, including age, sex assigned at birth, gender identity, race/ethnicity, past medical history, current medication use, smoking status, GATT dose, route of administration, duration of exposure, and time between last dose and study day. Participants were also invited to complete voluntary surveys, including the Perceived Stress Scale [32] and Patient Health Questionaire-9 (PHQ-9) Depression Survey [33]. All participants consumed a high salt diet consistent with typical Western food choices [34] (> 150 mmol sodium/day) for 3 days before the study day. A high-salt state was confirmed by 2nd morning void spot urine [35] using a potentiometric assay (Cobas 8000; Roche Diagnostics, Indianapolis, Indiana, USA). Participants arrived at the laboratory at 0800 h after an overnight fast. Participants were instructed to take their medications as per usual schedule. Those who consumed caffeine or alcohol, smoked tobacco, or used e-cigarettes and/or cannabis-derived products were asked to abstain for ≥ 8 h. Study visits were conducted by a Registered Nurse (DYS). Participants were studied in a quiet, temperature-controlled room.

### Exposure and Laboratory measurements

Samples for laboratory measurements were drawn in the morning of the study day, and analyzed by Alberta Precision Laboratories (Calgary, Alberta, Canada). Laboratory data included serum measures of hemoglobin A1c (colorimetric assay; Cobas c513; Roche Diagnostics, Indianapolis, Indiana, USA), fasting glucose (hexokinase-UV assay; Cobas 8000; Roche Diagnostics, Indianapolis, Indiana, USA), creatinine (enzymatic colorimetric assay utilizing creatininase; Cobas 8000; Roche Diagnostics, Indianapolis, Indiana, USA), as well as estradiol and progesterone (competitive chemiluminescent assay; Cobas 8000; Roche Diagnostics, Indianapolis, Indiana, USA). eGFR was calculated for each participant using the 2021 Chronic Kidney Disease Epidemiology Collaboration creatinine equation, where male was inputted as the sex covariate [36]. Spot urine samples were analyzed for quantification of both urine creatinine and urine albumin using Cobas 8000 and c701-c502 assays (Roche Diagnostics, Indianapolis, Indiana, USA), respectively, which were used to calculated uACR. LDL-c was estimated using the National Institutes of Health Research LDL-c equation [37], inputting serum total cholesterol, triglycerides, and high-density lipoprotein cholesterol, which were all measured using an enzymatic colorimetric assay (Cobas 8000; Roche Diagnostics, Indianapolis, Indiana, USA).

The exposure, total serum testosterone concentration, was measured by a standard-of-care competitive chemiluminescent immunoassay (Cobas 8000; Roche Diagnostics, Indianapolis, Indiana, USA) that has previously been shown to give results consistent to those measured by mass spectrometry in individuals on GATT[38]. Albumin concentration was determined using a bromocresol green assay (Cobas 8000; Roche Diagnostics, Indianapolis, Indiana, USA). Sex hormone binding globulin (SHBG) was measured by an immunoassay (Immulite 2000; Siemens Healthineers, Erlangen, Bavaria, Germany). Using total serum testosterone, albumin, and SHBG concentrations, free and bioavailable testosterone concentrations were calculated [39].

### Study outcomes: hemodynamic and arterial stiffness measures

The primary outcome was systolic blood pressure (SBP), as there is a strong association between incremental increases in normotensive blood pressure and cardiovascular risk, even in the absence of risk factors [40]. Blood pressure readings were taken in seated position after a minimum of 10 min of rest without speaking or sleeping. SBP measurements were recorded with an automatic recording device (Critikon DINAMAP ProCare Monitor, GE Medical Systems, Milwaukee, Wisconsin, USA) as per guidelines [41]. After discarding the first measure, the mean of two readings taken by the same Registered Nurse (DYS) were recorded. Carotid-radial pulse wave velocity (PWVcr) and aortic augmentation index (AIx) are validated measures of peripheral and central vascular stiffness, respectively, and are associated with adverse cardiovascular outcomes [42]. PWVcr and AIx were measured noninvasively in supine position on the participant’s right side with applanation tonometry (Millar Instruments, Houston, Texas, United States) and commercially available acquisition and analysis software (Version 8 SphygmoCor; AtCor Medical, Sydney, New South Wales, Australia) as previously described [43]. AIx was standardized to a heart of 75 beats per minute.

### Statistical analysis

Descriptive statistics were reported as medians with interquartile range (IQR) and range in this exploratory study. Univariable linear regression analyses were used to estimate the relationship between total and free serum testosterone concentration, and SBP and arterial stiffness. Linearity was assessed using visual inspection of an augmented component-plus-residual plot and the non-linearity check [44] command in Stata V.17.0 (StataCorp, College Station, Texas, USA). The Shapiro–Wilk [45], and Breusch–Pagan [46] tests were used to assess the normality and heteroscedasticity assumptions, respectively. The strength of each relationship was assessed through reporting of beta coefficients, 95% confidence intervals (CI), and p-values. All secondary analyses were determined post-hoc due to the exploratory nature of the study, and based on data availability, involving solely our primary outcome of SBP to minimize the possibility of committing a type 1 error. Participants were excluded from analyses when they had missing data on the exposure or outcome of interest. Statistical analyses were completed using Stata V.17.0 (StataCorp, College Station, Texas, USA). Significance was defined as p < 0.05.

## Results

### Baseline demographics

Study cohort demographics are displayed in Table 1**.** In total, 18 participants were included (men, n = 9; nonbinary, n = 9) with a median age of 28 (IQR: 13) years. A single participant did not complete their bloodwork, and their results are only included in the demographic data. The majority of participants self-identified as white and one participant was a tobacco smoker. All participants were normotensive. BMI ranged from ideal to obese measures, with the median BMI in the overweight range [47]. Participants’ Perceived Stress Scale median score was classified as moderate stress, and the median PHQ-9 Depression Survey score was consistent with mild depression. There were no differences in baseline parameters between men and nonbinary participants.

**Table 1:** Baseline characteristics

n = 18	
Age (years)	28 [13] (18, 50)
Sex assigned at birth Female	18 (100)
Gender identity Man Nonbinary	9 (50) 9 (50)
Race/ethnicity Hispanic/Latinx White	1 (6) 17 (94)
Current tobacco smoker, N (%) No Yes	17 (94) 1 (6)
Current cannabis and/or E-cigarette user* No Yes	12 (67) 6 (33)
BMI (kg/m^2^)	27 [9] (20, 49)
Abdominal Circumference (cm)	83 [18] (64, 129)
Hemoglobin A1c (%)	5.3 [0.6] (3.7, 5.7)
Fasting Glucose (mmol/L)	4.2 [0.6] (3.6, 5.9)
LDL-c (mmol/L)	2.3 [0.9] (1.6, 3.9)
Serum Total Testosterone (nmol/L)	17 [10] (6, 33)
Serum Free Testosterone (nmol/L)	0.4 [0.3] (0.2, 0.4)
Serum SHBG (nmol/L)	26 [21] (10, 53)
Serum Estradiol (pmol/L)	165 [85] (53, 1103)
Serum Progesterone (nmol/L)	0.9 [0.5] (0.3, 1.9)
Serum Albumin (g/L)	38 [3] (34, 42)
eGFR (mL/min/1.73m^2^)	108 [28] (79, 134)
uACR (mg/mmol)	0.5 [0.2] (0.2, 1.5)
SBP (mmHg)	113 [8] (102, 129)
DBP (mmHg)	66 [10] (55, 78)
Perceived Stress Scale	22 [4] (15, 28)
PHQ-9 Depression Survey	10 [7] (1, 20)

*Notes.* Values are reported as Median [IQR] (Range) or n (%). No statistically significant differences between men and non-binary study participants. *: Includes all cannabis-derived products. Abbreviations: BMI: Body Mass Index. DBP: Diastolic Blood Pressure. eGFR: Estimated Glomerular Filtration Rate. LDL-c: Low Density Lipoprotein Cholesterol. PHQ: Patient Health Questionnaire. SBP: Systolic Blood Pressure. SHBG: Sex Hormone Binding Globulin. Missing data: Fasting Glucose (n = 1); LDL (n = 1); Serum Total Testosterone (n = 1); Serum Free Testosterone (n = 2); SHBG (n = 2); Serum Estradiol (n = 1); Serum Progesterone (n = 1); Serum Albumin (n = 1); eGFR (n = 2); uACR (n = 2)

### Gender-affirming testosterone therapy characteristics

**Fig. 1:**
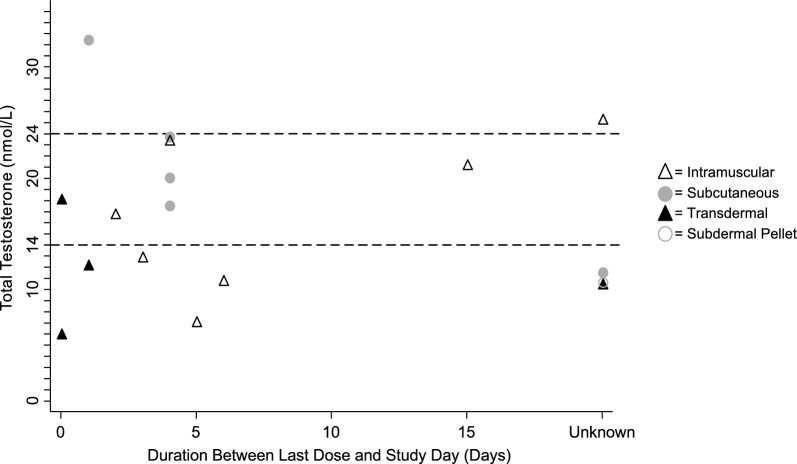
Time between last study day and corresponding total testosterone concentration of 17 transgender study participants. Results are stratified by route of administration. Dashed lines indicate reference range for total testosterone (14–24 nmol/L) suggested in the *Endocrine Treatment of Gender-Dysphoric/Gender-Incongruent Persons: An Endocrine Society Clinical Practice Guideline* [7, 9]

GATT characteristics are presented in Table 2. Three-quarters of participants used an injectable form of GATT, with the majority using an intramuscular route of administration. The median duration of GATT use was 4 years and ranged from 5 months to 7 years. Two participants used concomitant estrogen (n = 1 topical estrogen-based vaginal cream, n = 1 oral contraceptive). There were no significant differences between men and nonbinary participants. The total testosterone concentration of ten participants was outside of the recommended gender-affirming care reference range (14–24 nmol/L [7]) (Fig. 1).

**Table 2:** Gender-affirming testosterone therapy characteristics

n = 18	
Route of Administration Intramuscular Subcutaneous Transdermal Subdermal pellet	8 (44) 5 (28) 4 (22) 1 (6)
Defined daily dose (mg)	0.6 [0.6] (0.01, 1)
Duration of use (months)	48 [36] (5, 84)
Duration between last dose and study day (days)	4 [3] (0, 15)

*Notes.* Values are reported as Median [IQR] (Range) or n (%). No statistically significant differences between men and non-binary study participants. Missing Data: Defined Daily Dose (n = 4); Duration Between Last Dose and Study Day (n = 5)

### Serum testosterone concentration and cardiovascular health

When assessing the assumptions of linear regression, there was no evidence of violation of the normality and heteroscedasticity assumptions. However, total testosterone demonstrated a non-linear relationship with SBP. Fractional-polynomial transformations did not correct the violated assumptions, but transformation by squaring total serum testosterone did, and the estimated association is presented as such.

**Fig. 2:**
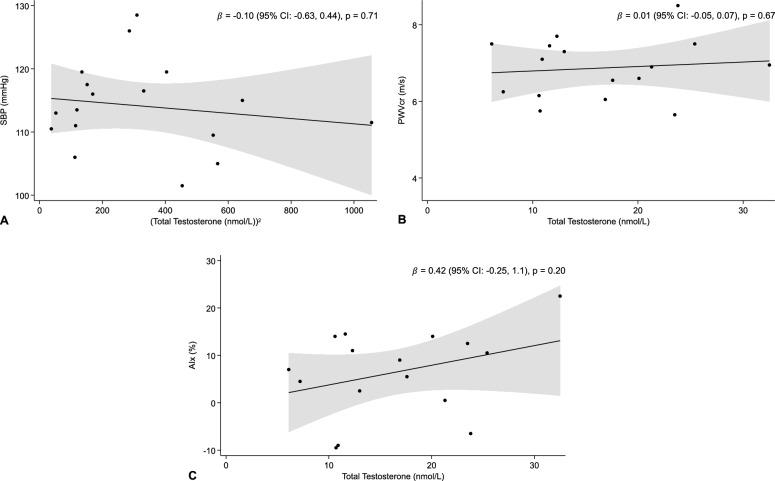
Association between total testosterone and **A** SBP (n = 17), **B** PWVcr (n = 16), **C** AIx (n = 16), with 95% confidence band. Total testosterone demonstrated a non-linear relationship with SBP. A square transformation was applied to correct the violated assumption. Abbreviations: AIx: Aortic Augmentation Index. CI: Confidence Interval. PWVcr: Carotid-Radial Pulse-Wave Velocity. SBP: Systolic Blood Pressure

**Fig. 3:**
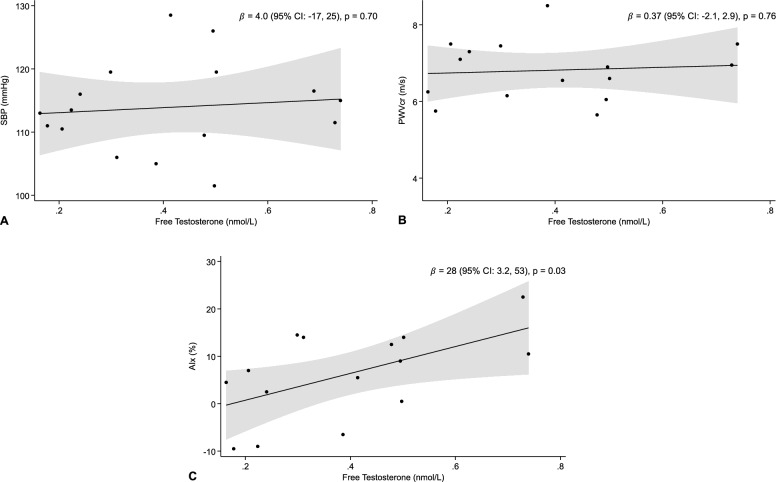
Association between free testosterone and (**A**) SBP (n = 16), (**B**) PWVcr (n = 15), (**C**) AIx (n = 15), with 95% confidence band. Abbreviations: AIx: Aortic Augmentation Index. CI: Confidence Interval. PWVcr: Carotid-Radial Pulse-Wave Velocity. SBP: Systolic Blood Pressure

No significant associations were observed between total serum testosterone concentration and SBP, PWVcr, or AIx (Fig. 2). While no significant associations were observed between free serum testosterone concentration and SBP or PWVcr, a significant positive association with AIx ($\beta$=28, p = 0.03) was demonstrated (Fig. 3). When assessing gender-identity as a potential effect modifier on the association between our exposures and primary outcome, no difference was identified between men and non-binary individuals (Table S1). Additional exploratory analyses showed no association between SHBG or other serum sex hormone concentrations (bioavailable testosterone, estradiol, testosterone-to-estradiol ratio, progesterone) and SBP (Table S2). Sensitivity analyses including only subcutaneous and/or intramuscular GATT users (n = 13), excluding participants using estrogen-based compounds in addition to GATT (n = 2), and excluding current tobacco smokers (n = 1) did not appreciably change the results (Table S3).

## Discussion

In this study, we estimated the association between serum testosterone concentration and markers of cardiovascular health in healthy transgender individuals using GATT. Our key finding was that serum total testosterone was not associated with SBP or measures of arterial stiffness, both validated markers of cardiovascular health [40, 42]. Taken together, these results suggest that in healthy transgender individuals treated with GATT, serum testosterone concentrations may not be associated with measures of cardiovascular health. However, these results need to be interpreted with caution given the limited sample size.

The associations between endogenous serum testosterone concentrations and blood pressure in different populations are conflicting. A recent meta-analysis [15] of prospective cohort studies with at least five years of follow-up reported that community-dwelling men with very low serum testosterone concentrations (< 5.3 nmol/L [< 153 ng/dL]) had a significantly increased risk of cardiovascular death. However, no association between serum testosterone concentration and incident cardiovascular events was observed. In contrast, a population-based study of 1428 women [21] reported a positive association between serum total testosterone and blood pressure in both cross-sectional and longitudinal analyses. In a nested prospective cohort study of 4716 women not receiving oral contraceptives or hormone therapy [20] participants with serum testosterone concentrations in the top 5th percentile had a significantly greater risk of ischemic heart disease and death than those with concentrations falling between the 10th and 89th percentiles. Of note, a single measurement has been suggested to be fairly representative for the long-term hormonal milieu in men [48], but if or how testosterone concentrations change over time in women is less clear. Moreover, baseline mean (standard deviation (SD)) SBP among participants in both of these studies were similar to one another, at 130 [21] mmHg [21] and 137 [23] mmHg [20], but greater compared to participants in the present study. These differences across populations may partly reflect sex- and gender-related physiological testosterone effects on the vasculature [49], although this remains unexamined in the literature.

Interestingly, approximately half of individuals had total serum testosterone concentrations outside of clinically recommended references ranges, which are based on cisgender men [6, 7]. In the absence of evidence of harm with concentrations outside of this range, this may reflect the need to re-evaluate recommendations regarding target concentrations; this requires further research as previously highlighted [50]. Of note, free, but not total, testosterone demonstrated a significant association with AIx. In circulation, testosterone can be found bound to albumin or SHBG, which regulate its transport, metabolism, and distribution, but it also can be unbound [51]. According to the *Free Hormone Hypothesis* [52, 53], unbound, or free, but not bound, testosterone exhibits androgenic activity. Thus, total testosterone concentrations alone may not accurately reflect androgen activity, as it includes both bound and unbound testosterone, and if the majority is bound, its bioavailability may be limited [54]. In contrast, free testosterone, which is unbound and biologically active, may better capture androgenic effects. This distinction may be particularly relevant to vascular health, as testosterone has been shown to increase oxidative stress [55], induce apoptosis of vascular smooth muscle cells [56], and promote vasoconstriction [57]—all of which may be contributing to impaired AIx. Given free testosterone is likely only exerting androgenic effects on vascular tissues, this may in part explain the observed association with free, but not total, testosterone. The measurement and monitoring of total testosterone concentration in transgender individuals using GATT is recommended [7]. However, our results may indicate the potential importance of also monitoring free testosterone concentrations. Nonetheless, this hypothesis is speculative and warrants further investigation.

This study has limitations. Our sample size was limited, and recruitment occurred through snowball and convenience sampling, which may have introduced selection bias and constrained our ability to identify significant associations. Only healthy individuals using GATT were included in our study population; our results may thus not be generalizable to individuals living with chronic conditions. However, by studying only healthy participants in a controlled environment we were able to minimize confounding factors when examining the association between serum testosterone concentrations and measures of cardiovascular health. Next, the cross-sectional study design limits conclusions regarding causality, although the objective of our study was to examine any association between serum testosterone concentrations and cardiovascular health with GATT use. Finally, GATT is meant to be a lifelong therapy, and whether the results of this study persist after prolonged exposure to exogenous testosterone is unknown. Due to the limited sample size, we were unable to stratify results by route of administration, and account for age at GATT initiation or GATT dose in our models, highlighting the need to interpret our results with caution. We also did not account for duration of GATT use, which spanned from 5 months to 7 years in this study. Duration of use has varied significantly in previous studies investigating the relationship between GATT use and cardiovascular health, ranging from 3 months to 49 years [3]. However, sensitivity analyses by type of GATT did not alter our study results, and a previous cohort study reported stabilization of blood pressure after 4 months of GATT use [30], suggesting that the lack of association between serum total testosterone concentrations and measures of vascular health noted in our study may persist with time, although further longitudinal study is required [58].

## Perspectives and significance

A previous study has shown an association between GATT use and increased blood pressure in transgender individuals, with the presumed mechanism being exposure to exogenous testosterone and a subsequent increase in SBP [30]. Interestingly, participants’ mean (SD) SBP was 124 [11] mmHg at a follow-up of 46–57 months, which differs from the median SBP of participants in the present study (113 mmHg). Differences in observed SBP may be in part due to the exclusion of individuals living with hypertension in this study, pointing towards the need to examine GATT use in hypertensive and non-hypertensive transgender individuals. Moreover, in keeping with our findings of no association between increasing serum total testosterone concentrations with exposure to exogenous testosterone and cardiovascular risk, a recent systematic review of 17 short-term randomized controlled trials in men showed no association between testosterone therapy and cardiovascular risk [59]. A more recent randomized controlled trial of 5246 hypogonadal men treated with either transdermal testosterone therapy or placebo [60] demonstrated no differences in major adverse cardiovascular events between groups after 48 months. Taken together, the safety of GATT use may be similar to that of testosterone therapy use in hypogonadal men. However, further research is required, which should aim to recruit a larger sample, refine exposure characterization, accounting for GATT dose, route of administration, and pharmacokinetics, and collect longitudinal data to improve causal interpretation.

## Conclusions

This community-based cross-sectional study did not identify an association between serum total testosterone concentration and markers of cardiovascular health in individuals treated with GATT. Given the positive associations between physical and psychosocial well-being of transgender individuals with gender-affirming hormone therapy use [61–63], and ongoing efforts to achieve in equity in hypertension [64], our findings may have important implications in the care of individuals using GATT. However, large-scale prospective studies will need to be performed before recommendations regarding clinical practice can be made.

## Supplementary Information

Additional file 1.

## Data Availability

De-identified data are available from the corresponding author on reasonable request.
