# Supplementary material for: Association between serum testosterone and measures of cardiovascular health among transgender individuals using gender-affirming testosterone therapy: a cross-sectional study
Source: Biol Sex Differ. 2025 Jun 17;16:44. doi: 10.1186/s13293-025-00726-3 (PMC12172241; doi:10.1186/s13293-025-00726-3)
Supplement: Supplementary file 1 — Additional file 1. [file 13293_2025_726_MOESM1_ESM.pdf]

Table of Contents

Table S1. Results of assessment of gender-identity as an effect modifier 1

Table S2. Estimated association between additional sex hormone markers and concentrations, and systolic blood pressure. 2

Table S3. Sensitivity analyses conducted when estimating associations between serum testosterone concentrations and systolic blood pressure. 3

Appendix A. STROBE Statement. 4

Table S1. Results of assessment of gender-identity as an effect modifier.

Estimated Association	β_{Men} (95% CI)	p-value	$\beta_{\text{Nonbinary}}$ (95% CI)	p-value	$\beta_{\text{Interaction}}$ (95% CI)	p-value
Total Testosterone and SBP	-0.09 (-0.65, 0.47)	0.74	-0.02 (-0.82, 0.78)	0.95	-0.06 (-0.57, 0.44)	0.79
Free Testosterone and SBP	4.0 (-18, 26)	0.70	7.6 (-23, 38)	0.60	-3.6 (-24, 17)	0.72

Notes. Participants included in each analysis: Total testosterone (n=17). Free Testosterone (n=16). Abbreviations: CI: Confidence Interval. SBP: Systolic Blood Pressure.

Table S2. Estimated association between additional sex hormone markers and concentrations, and systolic blood pressure.

Variable of Interest	SBP (mmHg)	
	**β (95% CI)**	**p-value**
**SHBG (nmol/L)**	-0.18 (-0.49, 0.12)	0.23
**Bioavailable Testosterone (nmol/L)**	0.24 (-0.77, 1.3)	0.62
**Estradiol (pmol/L)**	0.00 (-0.02, 0.02)	0.67
**Testosterone:Estradiol Ratio**	0.02 (-0.04, 0.08)	0.52
**Progesterone (µg/L)**	0.64 (-8.0, 9.3)	0.88

Notes. Participants included in each analysis: SHBG (n=16). Bioavailable Testosterone (n=16). Estradiol (n=17). Testosterone:Estradiol Ratio (n=17). Progresterone (n=17). Abbreviations: CI: Confidence Interval. SBP: Systolic Blood Pressure. SHBG: Sex Hormone Binding Globulin.

Table S3. Sensitivity analyses conducted when estimating associations between serum testosterone concentrations and systolic blood pressure.

Sensitivity Analysis	Variable of Interest	SBP (mmHg)	
**Including Subcutaneous and/or Intramuscular GATT**		**β (95% CI)**	**p-value**
	**Total Testosterone (nmol/L)**	-0.34 (-1.1, 0.39)	0.33
	**Free Testosterone (nmol/L)**	-2.4 (-32, 28)	0.86
**Excluding Estrogen-Based Compound Use**		**β (95% CI)**	**p-value**
	**Total Testosterone (nmol/L)**	-0.04 (-0.61, 0.53)	0.89
	**Free Testosterone (nmol/L)**	2.7 (-19, 25)	0.79
**Excluding Tobacco smokers**		**β (95% CI)**	**p-value**
	**Total Testosterone (nmol/L)**	-0.10 (-0.67, 0.46)	0.70
	**Free Testosterone (nmol/L)**	4.0 (-18, 27)	0.71

Notes. Abbreviations: GATT: Gender-Affirming Testosterone Therapy. CI: Confidence Interval. SBP: Systolic Blood Pressure.

Appendix A. STROBE Statement. Checklist of items that should be included in reports of cross-sectional studies.

	Item No	Recommendation	Page No
**Title and abstract**	1	(*a*) Indicate the study's design with a commonly used term in the title or the abstract	1
		(*b*) Provide in the abstract an informative and balanced summary of what was done and what was found	2-3
**Introduction**			
Background/rationale	2	Explain the scientific background and rationale for the investigation being reported	5-6
Objectives	3	State specific objectives, including any prespecified hypotheses	6
**Methods**			
Study design	4	Present key elements of study design early in the paper	7-8
Setting	5	Describe the setting, locations, and relevant dates, including periods of recruitment, exposure, follow-up, and data collection	7
Participants	6	(*a*) Give the eligibility criteria, and the sources and methods of selection of participants	7
Variables	7	Clearly define all outcomes, exposures, predictors, potential confounders, and effect modifiers. Give diagnostic criteria, if applicable	8-10
Data sources/ measurement	8	For each variable of interest, give sources of data and details of methods of assessment (measurement). Describe comparability of assessment methods if there is more than one group	8-10
Bias	9	Describe any efforts to address potential sources of bias	11
Study size	10	Explain how the study size was arrived at	7
Quantitative variables	11	Explain how quantitative variables were handled in the analyses. If applicable, describe which groupings were chosen and why	11
Statistical methods	12	(*a*) Describe all statistical methods, including those used to control for confounding	11
		(*b*) Describe any methods used to examine subgroups and interactions	11
		(*c*) Explain how missing data were addressed	11
		(*d*) If applicable, describe analytical methods taking account of sampling strategy	N/A
		(*e*) Describe any sensitivity analyses	11
**Results**			
Participants	13	(a) Report numbers of individuals at each stage of study—eg numbers potentially eligible, examined for eligibility, confirmed eligible, included in the study, completing follow-up, and analysed	11

		(b) Give reasons for non-participation at each stage	N/A
		(c) Consider use of a flow diagram	N/A
Descriptive data	14	(a) Give characteristics of study participants (eg demographic, clinical, social) and information on exposures and potential confounders	11-12
		(b) Indicate number of participants with missing data for each variable of interest	Table 1 & 2
Outcome data	15	Report numbers of outcome events or summary measures	11-12
Main results	16	(*a*) Give unadjusted estimates and, if applicable, confounder-adjusted estimates and their precision (eg, 95% confidence interval). Make clear which confounders were adjusted for and why they were included	13
		(*b*) Report category boundaries when continuous variables were categorized	Table 1 & 2
		(*c*) If relevant, consider translating estimates of relative risk into absolute risk for a meaningful time period	N/A
Other analyses	17	Report other analyses done—eg analyses of subgroups and interactions, and sensitivity analyses	13
**Discussion**			
Key results	18	Summarise key results with reference to study objectives	14
Limitations	19	Discuss limitations of the study, taking into account sources of potential bias or imprecision. Discuss both direction and magnitude of any potential bias	16-17
Interpretation	20	Give a cautious overall interpretation of results considering objectives, limitations, multiplicity of analyses, results from similar studies, and other relevant evidence	14-16
Generalisability	21	Discuss the generalisability (external validity) of the study results	17-18
**Other information**			
Funding	22	Give the source of funding and the role of the funders for the present study and, if applicable, for the original study on which the present article is based	19-20
